# Mechanics of the IL2RA Gene Activation Revealed by Modeling and Atomic Force Microscopy

**DOI:** 10.1371/journal.pone.0018811

**Published:** 2011-04-13

**Authors:** Pascale Milani, Monique Marilley, Albert Sanchez-Sevilla, Jean Imbert, Cédric Vaillant, Françoise Argoul, Jean-Marc Egly, José Rocca-Serra, Alain Arneodo

**Affiliations:** 1 RGFCP EA 3290, Faculté de Médecine, Marseille, France; 2 Université de Lyon, Lyon, France; 3 Laboratoire Joliot-Curie and Laboratoire de Physique, CNRS, Ecole Normale Supérieure de Lyon, Lyon, France; 4 U928 Inserm, TAGC, Marseille, France; 5 Université de la Méditerranée, Marseille, France; 6 Institut de Génétique et de Biologie Moléculaire et Cellulaire, CNRS/INSERM/Université de Strasbourg, Illkirch, France; Ludwig-Maximilians-Universität München, Germany

## Abstract

Transcription implies recruitment of RNA polymerase II and transcription factors (TFs) by DNA melting near transcription start site (TSS). Combining atomic force microscopy and computer modeling, we investigate the structural and dynamical properties of the IL2RA promoter and identify an intrinsically negative supercoil in the PRRII region (containing Elf-1 and HMGA1 binding sites), located upstream of a curved DNA region encompassing TSS. Conformational changes, evidenced by time-lapse studies, result in the progressive positioning of curvature apex towards the TSS, likely facilitating local DNA melting. In vitro assays confirm specific binding of the General Transcription Factors (GTFs) TBP and TFIIB over TATA-TSS position, where an inhibitory nucleosome prevented preinitiation complex (PIC) formation and uncontrolled DNA melting. These findings represent a substantial advance showing, first, that the structural properties of the IL2RA promoter are encoded in the DNA sequence and second, that during the initiation process DNA conformation is dynamic and not static.

## Introduction

RNA polymerase II-dependent transcription is initiated at a promoter, with a core region able to sustain basal RNA synthesis. Initiating transcription implies the binding of GTFs and RNA polymerase II on the promoter as well as the localized melting of DNA in the TSS region [1]–[3]. Initiation thus requires that the DNA structure facilitates the binding of these factors as well as the appropriate DNA melting. Special attention has been devoted to the DNA structure in the promoter region as a plausible physical support for promoter activation [4]–[8]. Indeed, since the discovery of intrinsically curved DNA [9], many experiments have established that the local sequence-dependent physical (structural, mechanical) properties of the DNA double helix are one of the major ingredients in DNA-protein interactions [10], [11]. In particular, the energy cost for nucleosome formation presents a sequence-dependent contribution which may influence the positioning of nucleosomes, and in turn the access of TFs to their target sites thereby contributing to gene expression regulation [12], [13]. Recent studies have shown that the DNA sequence actually codes for high-energy barriers that impair nucleosome formation [14]–[16], and condition the collective assembly of neighboring nucleosomes according to equilibrium statistical ordering principles [16]–[19]. These genomic excluding-energy-barriers are highly predictive of the nucleosome-free regions (NFRs) observed *in vitro* and *in vivo* at the promoter of genes that are constitutively expressed [18]–[21]. Alternatively, the relative positioning of these energy barriers with respect to the TSS can also condition an inhibitory nucleosomal chromatin environment that represses gene-transcription [19].

Among the various experimental techniques that have been used to investigate DNA binding and flexibility, atomic force microscopy (AFM) has proved to be very efficient. It can be used to estimate the persistence length of DNA via end-to-end distance measurements [22]–[26], but more interestingly it can give access, via thermodynamical averaging over a population of conformations, to both the intrinsic DNA curvature and bending flexibility with a surprisingly good resolution of 2–3 double-helix pitches [23], [24], [27]. AFM imaging has also been applied to characterize the chromatin structure at the nanoscale level [28]. Nucleosome assembly has been visualized, mainly in air, and in the specific context of positioning sequences (e.g. *Xenopus* 5S rDNA and 601 DNA sequences) and arrays of concatenated repetitions of these sequences [28]–[31]. Only recently, AFM imaging in liquid has provided new insight into the role of the DNA sequence on the collective organization of nucleosomes along the eukaryotic chromatin fiber [19], [32]. Single-molecule time-lapse AFM approach has also proved to be capable of detecting the dynamics of DNA conformational changes [33], [34], of telomeric nucleosomes [35], of unwrapping of DNA from the histone core in the absence of ATP-dependent chromatin remodelers [36] and of molecular systems involved in transcription [37] and replication [38].

Here we combined AFM imaging in liquid and physical DNA modeling to investigate the structural and dynamical DNA properties, nucleosome assembly and TF binding in the promoter region of the human IL2RA gene which plays a major role in the control of the immune system response [39]. We observed a very specific DNA structural organization linked to the IL2RA gene promoter sequence [40] that conditions the positioning of a nucleosome on the TATA box and TSS region as previously observed *in vivo* in unstimulated T-cells [41]. Besides preventing the binding of TBP and TFIIB and in turn PIC formation on the TATA box, this repressive nucleosome positioning likely protects from uncontrolled DNA melting in the TSS region. Time-lapse studies also revealed characteristic conformation changes under local DNA unwinding constraints that eventually facilitate DNA melting at the TSS. We elaborate on a model that incorporates these structural and dynamical DNA features of the IL2RA gene promoter region in its functional chromatin context. In particular we discuss the possible contribution of this constitutive repressive chromatin environment to transcription activation by favoring multiple HMGA1 binding, nucleosome remodeling and DNA unwinding at the TSS.

## Materials and Methods

### DNA samples and proteins preparation

**Figure 1:**
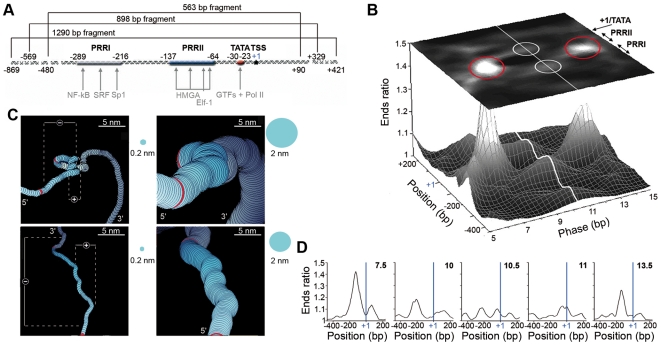
Theoretical modeling of the DNA structure of the IL2RA gene promoter. (A) Schematic representation of the 1290 bp human IL2RA gene fragment between nucleotides −869 bp and +421 bp, including the positive regulatory regions PRRI and PRRII, the TATA box (TACTTAAA) and the TSS (+1). The locations of the 898 bp and 563 bp fragments are also indicated. Positions of major TF binding sites are shown by grey arrows. (B) Theoretical analysis of the 1290 bp IL2RA sequence. Diagrammatic (upper plane) and spectral (lower graph) representation of the curvature as estimated by the Ends ratio at a given position for a given phasing pitch value (Materials and Methods). Red circles correspond to right-handed and left-handed supercoiled DNA. White circles and white line correspond to theoretically predicted and experimentally detected 2D curvatures [40]. (C) 3D modeling of the PRRII region sequence (delimited by the red labels). Left panels show the DNA double helix axis trajectory (thickness = 0.2 nm) from two different orientations. The negative and positive supercoil locations are specified. Right panels display the corresponding DNA molecule at physical scale (thickness = 2 nm). (D) Curvature profiles predicted by modeling the DNA fragment under over-twisting (pitch values 7.5 and 10 bp) or under-twisting (pitch values 11 and 13.5 bp), as compared to the 10.5 bp canonical reference value. The vertical blue line indicates the TSS (+1) position. In (b,d): window size is 150 bp, analyzed phasing pitch varies from 5 to 15 bp with step 1 bp.

Three DNA fragments of length 563 bp, 898 bp and 1290 bp containing the 5′ proximal promoter region (Figure 1A) of the IL2RA human gene (EMBL:U57613, 12 October 2001) were obtained by PCR amplification of human placenta genomic DNA. Amplifications were performed using the Taq PCR Master Mix Kit (Qiagen) (for a list of primers, see Table S1). Mixtures were run on a 1% agarose gel and the isolated DNA fragments were extracted from the corresponding gel slice by using a MinElute® Gel Extraction Kit (Qiagen). The 976 bp pBR DNA fragment used as a negative control was obtained by EcoRI and NruI digestion of the pBR322 plasmid, separated by 1% agarose gel electrophoresis and purified using a Sephaglass kit (Amersham Pharmacia Biotech).

Full-length recombinant histone proteins of *Xenopus laevis* were over-expressed in bacteria and purified as previously experienced [19]. Full-length recombinant human TBP and TFIIB proteins were produced and purified as previously described [40]. HMGA1a protein (referred to as HMGA1 for simplicity in this report) is a kind gift of A. Benecke (Eilebrecht, S., Brysbaert, G., Wegert, T., Urlaub, H., Benecke, A. & Benecke, B.J. 7SK small nuclear RNA is direct affector of HMGA1 function in transcription. In preparation).

### DNA-TPB-TFIIB interaction

DNA and protein complexes were assembled using the 563 bp IL2RA DNA fragment in a final volume of 10 µl, with reaction mixture containing 10 mM DNA fragment, 5 µl of binding buffer (10 mM Tris-HCl, pH 7.5, 10 mM MgCl_2_, 100 mM NaCl, 10% Glycerol). Human proteins were used in the following amounts: TBP 15 g, TFIIB 125 ng. The reaction mixtures were incubated at 25°C for 30 min. The formation of complexes was controlled by gel electrophoresis (1.5% agarose) (Figure S1A) and AFM imaging.

### Nucleosome reconstitution

Nucleosome reconstitution was performed by the salt dialysis procedure. The reaction was stopped in TE buffer (10 mM Tris-HCl, pH 7.4, 1 mM EDTA) and 10 mM NaCl. Nucleosomes were reconstituted with a histone/DNA ratio of 1/0.8. The formation of NCP was controlled by gel electrophoresis (1.5% agarose) (data not shown) and AFM imaging [19].

### HMGA1-DNA complexes

The isolated 898 bp IL2RA fragment (1.5 pmol) was incubated with increasing amounts of HMGA1 in 18 µl of binding buffer (17 mM Tris, pH 7.8, 41 mM NaCl, and 1.7 mM EDTA). After incubation for 30 min room temperature, the formation of complexes was controlled by gel electrophoresis (6.5% polyacrylamide in 0.25X TBE) (Figure S1B). For AFM imaging we used a molar ratio of HMGA to DNA of 3∶1.

### AFM imaging

AFM imaging was performed in solution by using a Nanoscope IIIa microscope (Veeco/Digital Instruments, Santa Barbara, CA) equipped with a type-E scanner and in tapping mode [40]. Freshly cleaved mica (muscovite mica, grade V-I, SP1) served as a support for sample adsorption. DNA preparations, nucleosome reconstitution solution and DNA-TBP-TFIIB reaction mixture were diluted in different buffers according to different experimental protocols. In the presence of doubly charged counter ions (Ni^2+^) in the solution, DNA molecules are able to equilibrate on the surface before being captured in a given conformation [22], [42]. For scanning in solution, we used the following buffer solutions:

- Buffer A: 10 mM Tris-HCl, 0.1 mM NiCl_2_, pH 7.9
- Buffer B: 10 mM Tris-HCl, 1 mM NiCl_2_, pH 7.9

For DNA imaging (Figure S2), DNA preparations were diluted (0.3 ng/µl) in buffer A. A 10 µl droplet of the solution was deposited onto freshly cleaved mica and incubated for two minutes. A 100 µl volume of buffer A was added in the liquid cell prior to imaging. For time-lapse studies, images were registered and the direction of scanning was from top to bottom. The size of the window was 1×0.25 µm and the scanning frequency 3.38 Hz, which gave one image every 37 seconds.

For DNA-TBP-TFIIB complexes, nucleosomes, and HMGA1-DNA complexes imaging, reaction mixtures were diluted ∼4-fold in the buffer B. A 10 µl droplet of the solution was deposited onto freshly cleaved mica and incubated for two minutes. A 100 µl volume of buffer B was added in the liquid cell prior to imaging. AFM images were recorded from 1×1 µm or 2×2 µm frames, at a line scan rate of 2.18 Hz and a resolution of 512×512 pixels.

For all scans, commercial silicon nitride probes (type NP-S, Veeco Instruments) were used at a drive frequency of 8–9.5 kHz at ambient temperature.

### Image analysis

Image treatment and analysis were performed with the “Scanning Adventure” software [27]. Briefly, line-by-line second-order flattening was first applied, followed by thresholding and filtering. Zooms on individual DNA fragments, nucleosomes or DNA-TBP-TFIIB complexes were achieved and the DNA molecule path was skeletonized; then topographical analysis and length measurements were carried out (Figure S3). The results enabled a measurement of (i) the height variation along the DNA molecules which allowed us to orient the DNA fragment [19] (Figure S3), (ii) the DNA-protein complexes height (Figure S4) and (iii) the mapping of the nucleosome and TBP-TFIIB positions along the DNA fragments. From the measurement of the length of free DNA outside the nucleosome, namely L_+_ (resp. L_−_) for the longest (resp. shortest) arm, and in turn of the DNA complexed into nucleosome L_c_  =  L - (L _+_ + L _−_), where L is the total length of the considered DNA fragment, we positioned the dyad on this fragment by adding L_c_/2 to either L_+_ or L_−_ as previously described [19]. The position of the TBP-TFIIB-DNA complex was determined as corresponding to the local height maximum along the DNA skeleton. The same procedure was applied for the HMGA1-DNA complex. Positions and lengths were measured in nm and converted in bp with the approximate rise of 0.36 nm per bp [27].

For time-lapse images analysis, DNA conformational changes were followed on successive scans of the same molecule by calculating the Ends ratio along the molecule for each scan, with a 150 bp window and a 1 bp step. The Ends ratio is a measurement of the DNA curvature as given by the ratio: (length along the molecule)/(distance between the two ends of the DNA segment) in a selected window. These changes were registered in series of about 10 images on different molecules.

### DNA modeling

#### DNA duplex stability

The thermodynamic library [43] characterizing all 10 Watson-Crick nearest-neighbor interactions in DNA was used to calculate DNA duplex stability ΔG. These thermodynamic data provide an experimental basis for predicting the stability of any DNA duplex region by inspection of its primary sequence. Each calculated value takes into account the contribution of the surrounding nucleotides. We used the MAPall software [5] (previously PACS DNA), with a 150 bp window and a 1 bp step. All calculations were carried out with parameters and conditions (1 M NaCl, 25°C and pH 7) previously used by SantaLucia *et al* [43].

#### Curvature analysis and superhelix detection

The DNA path was calculated as the result of one translation and three rotations (roll, tilt and twist) at each nucleotide step using Bolshoy *et al.* values [44]. This DNA coding table was previously shown to provide very good fit of experimental DNA data from AFM analysis in liquid [40]. For superhelix detection, we used a method based on computer modeling allowing the detection of a left-handed (negative) as well as a right-handed (positive) superhelical organization of DNA encoded by the nucleotide sequence [40], [45]. The superhelical structure was made apparent by calculating the 3D DNA trajectory at different pitch values. When properly phased, the intrinsically curved elements make the DNA double helix path curved in a 2D plane. The coordinates calculated from the 3D trajectory of the helix axis were used to compute the Ends ratio in a 2D-curvature map. The phasing pitch value was expressed as a number of base pairs per turn.

## Results

### Theoretical modeling: prediction of the DNA structure of the IL2RA promoter

We investigated the 1290 bp 5′ proximal promoter region of the IL2RA human gene [39] (Figure 1A), that includes: (i) the TSS, (ii) the core promoter with the TATA box (TACTTAAA), (iii) two positive regulatory regions, PRRI and PRRII, lying respectively at position −289/−216 bp and −137/−64 bp upstream of the major TSS. PRRI contains binding sites for sequence-specific TFs, NF-κB, SRF and Sp1, whereas PRRII encompasses three binding sites for the architectural factor HMGA1 and one for Elf-1, a lymphoid-specific protein that belongs to the ETS TF family. According to the importance of these two elements for regulating inducible transcription of the IL2RA gene [39], our aim was to elucidate the possible contribution of supercoiled DNA structures to the intrinsic repression of this gene as well as their actual role in its activation. As noticed earlier [9], the periodic distribution of A/T stretches participates in determining the shape of bent DNA. If the periodicity is equal to the DNA canonical pitch ∼10.5 bp, DNA forms an easily detectable planar curve. However, if this periodicity is significantly different, then DNA forms either a right- or a left-handed 3D superhelix which turns out to be difficult to detect in practice. To unravel the presence of such supercoiled structures, we used a method based on computer modeling of the DNA trajectory using the 16 dinucleotide steps [44], [45]. Indeed, by artificially “twisting” (*i.e*. decreasing the phasing pitch) or “untwisting” (*i.e.* increasing the phasing pitch) the molecule, it is possible to detect a left- or a right-handed supercoil respectively by making them appear as a 2D bent curve. When reconstructing the curvature landscape (Figure 1B) from the successive Ends ratio profiles (Materials and Methods) obtained along the IL2RA promoter for increasing pitch values from 5 bp to 15 bp (Figure 1D), we indentified two new important conformational characteristics of the promoter. Besides the detected 2D curvatures (white circles on the grey-coded curvature map, Figure 1B, upper plane) identified on the Ends ratio profile for the canonical 10.5 bp pitch value, two Ends ratio peaks were obtained for pitch values ∼7.5 bp and 13.5 bp as the signature of negative and positive supercoiled DNA respectively (red circles on the grey-coded curvature map, Figure 1B, upper plane). Importantly, both these negative and positive supercoils co-map with PRRII [46]. These results were substantiated when using the MAPall software [5] to represent the 3D path of the DNA molecule in the vicinity of the PRRII region (Figure 1C). Two different supercoils coexist: a large amplitude, negative one which encloses a smaller positive one. This second one is hardly visible when the diameter of the double-helix is represented at the physical scale (Figure 1C, right panels); it becomes clear when focusing on the double-helix axis only (Figure 1C, left panels).

### AFM imaging of PRRII DNA supercoiling

**Figure 2:**
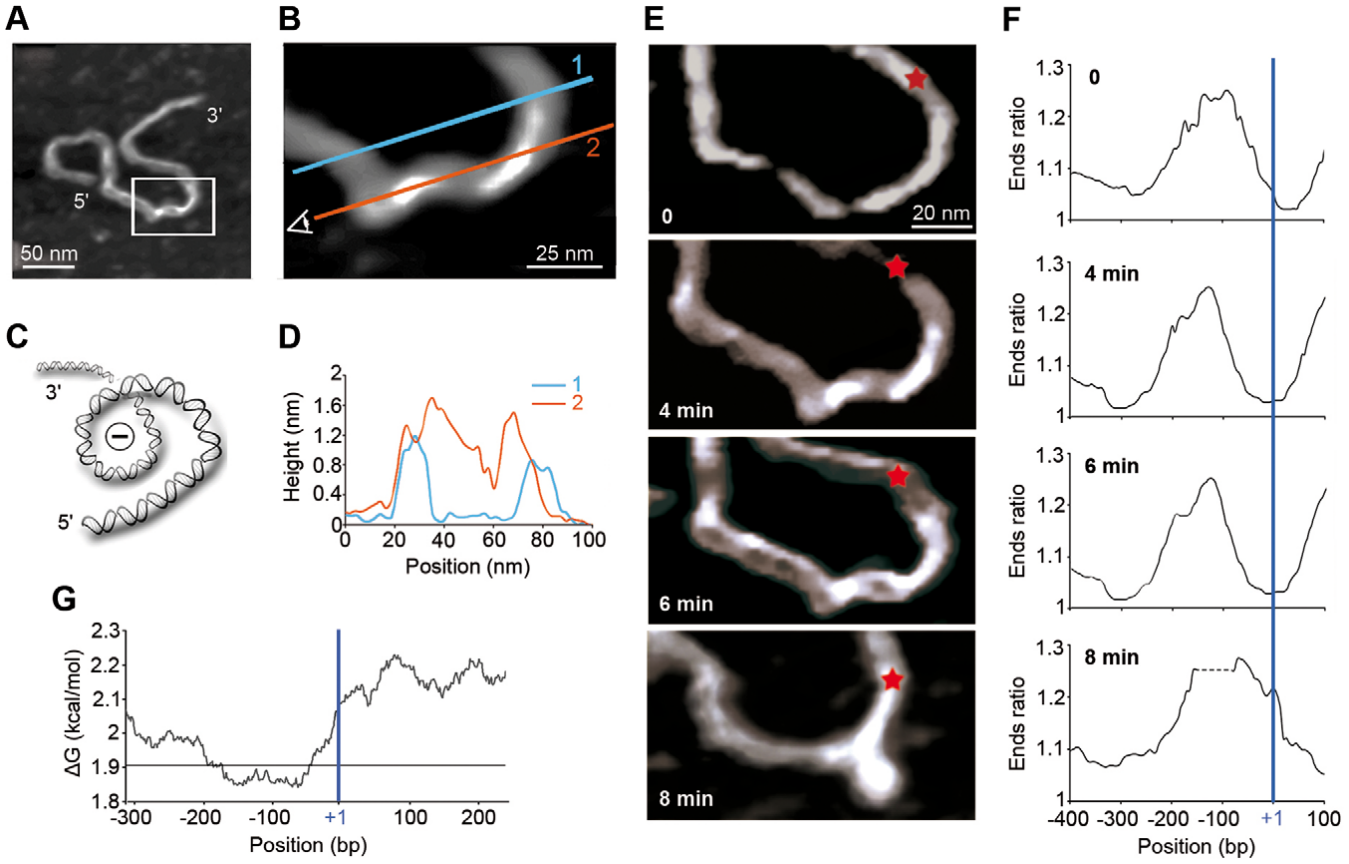
AFM imaging of the IL2RA gene promoter. (A) AFM image of the 1290 bp IL2RA fragment presenting a supercoiled structure. (B) Zoom on the region containing the supercoil, in the white rectangle in panel (A). Grey coding of topographic variations allows the visualization of the 3D shape of the structure. Sections 1 (blue line) and 2 (red line) correspond to the height measurements displayed in panel (D). (C) Schematic drawing of the negative supercoiled structure observed along the red vision axis 2 in panel (b). (D) Height measurements along the red and blue lines in panel (B). The supercoil summits are over 1.5 nm, whereas the double helix height in liquid ranges between 0.8 and 1.2 nm. (E) Time lapse AFM images of this fragment in the enlarged white box in (A) at 0, 4 min, 6 min and 8 min after beginning the observation. The position of the TSS is indicated by a red star. At 4 min, a negative supercoil was observed. A loop was formed out of the supercoiled region after 8 min. (F) Corresponding curvature profiles, expressed as Ends ratios (Materials and Methods), along the fragment (window 150 bp, step 1 bp). The position of the TSS (+1) is indicated by a vertical blue line. (G) ΔG variation profile along the 1290 bp IL2RA fragment. The horizontal line is the mean ΔG value over the whole gene sequence.

AFM imaging in liquid (Materials and Methods) allowed us to validate the theoretical prediction of the presence of an intrinsic supercoiled structure in the IL2RA promoter region (Figure 2A and Figure S2) and of its handedness as left-handed, *i.e.* negative (Figure 2C) as spectacularly revealed when zooming on the corresponding region (Figure 2B). From a quantitative point of view, the height profiles obtained along two different vision axis exhibit maxima as high as 1.4–1.6 nm along the supercoil that significantly exceed the characteristic ∼0.9–1.1 nm height of the double-helix lying on the mica surface (Figure 2D). Note that the standard deviation of height measurements from 100 molecules is fairly constant (∼0.3 nm) along the entire molecule trajectory (Figure S5E). After orientating the DNA molecule (Material and Methods and Figure S3), the supercoil was found to be located between positions −160±20 bp and −70±20 bp from the TSS, confirming its co-localization with the PRRII element. Importantly, this supercoil was robustly observed on the 1290 bp DNA fragment but also on the shortest fragment of length 563 bp (figure S2F and G). Furthermore, when using the 976 bp pBR DNA fragment as a control (Materials and Methods), no evidence of 3D supercoiled structure was ever obtained (Figure S5A, C-E), in agreement with theoretical modeling (Figure S5B). This experimental AFM study thus demonstrated the existence of a left-handed supercoiled structure in the PRRII region. However, to detect the presence of a small right-handed supercoiled structure, as also predicted by theoretical modeling (Figure 1), a higher resolution of the AFM tip needs to be achieved.

### Time-lapse AFM analysis of DNA structural changes

AFM observations made in liquid allow the tracking of DNA conformational changes in time, provided conditions for low adhesion to the support are satisfied. In such ionic conditions, the molecules explored different conformations although interactions with the mica surface remained sufficient to maintain their local 2D confinement necessary for AFM imaging [19], [40]. In addition, the scanning frequency was increased so that we could register one image of the same molecule every 37 seconds. We produced 3 movies that are available online (Videos S1, S2 and S3). As seen in selected frames (Figure 2E, and Figures S6 and S7), all these molecules exhibit a left-handed supercoiled structure at the same location as theoretically predicted (Figure 1).

**Figure 3:**
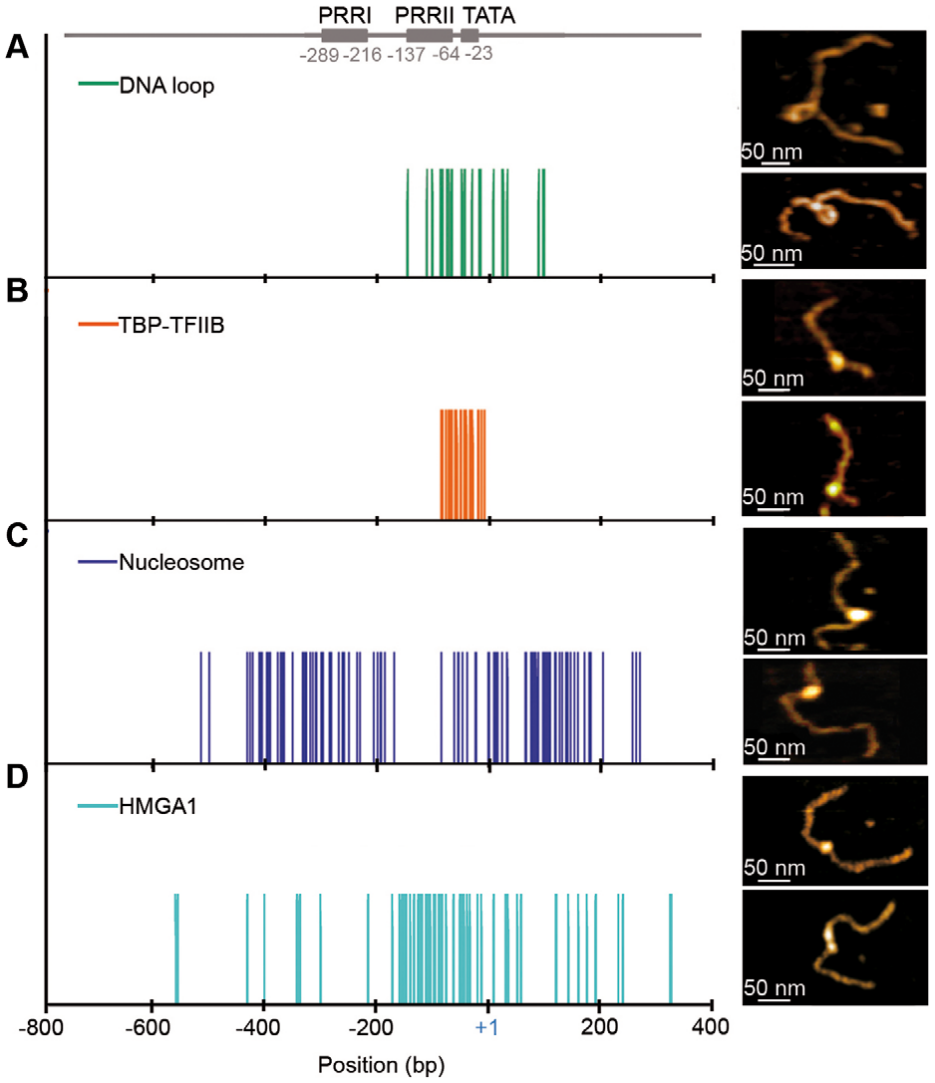
AFM imaging of DNA loop, GTF, nucleosome and HMGA1 positioning on the IL2RA gene promoter. (A) AFM imaging in liquid of DNA loop positioning along the 1290 bp IL2RA fragment (upper right panel) and the 898 bp IL2RA fragment (lower right panel) and statistical analysis (left panel) of their positioning given by the loop middle point (green vertical bars) from N = 21 AFM images. (B) AFM imaging of TBP-TFIIB positioning along the 563 bp IL2RA fragment (right panels) and statistical analysis (left panel) of their positioning (orange vertical bars) from N = 51 AFM images. (C) AFM imaging of mononucleosome positioning along the 898 bp IL2RA fragment (right panels) and statistical analysis (left panel) of dyad positioning (blue vertical bars) from N = 100 AFM images. (D) AFM imaging of HMGA1 positioning along the 898 bp IL2RA fragment (right panels) and statistical analysis (left panel) of dyad positioning (blue vertical bars) from N = 73 molecules.

To visualize structural modifications occurring at the IL2RA promoter, we zoomed on images taken at the beginning of the observation (T0) and successively 4, 6 and 8 minutes later (T4, T6 and T8 respectively). The supercoil encompassing the PRRII region definitely changed geometry (Figure 2E and Figures S6). Some negative overwinding of the supercoiled region progressively invaded the structure, eventually leading to the formation of a DNA loop (Figure 2E, bottom panel). Successive topographical profiles registered on the loop unambiguously demonstrated the left-handedness of this structure (Figure S8A–C). The analysis of as many as 21 of such left-handed DNA loops (Figure S9) assesses its positioning in the region extending from PRRII to the TATA box and TSS (Figure 3A). Despite some variability in size, these loops have a characteristic length ∼260 bp (∼94 nm) which is about twice the persistence length of B-DNA (∼50 nm) [25], [26].

To further analyze the consequences of these morphological changes on the curvature properties of these IL2RA molecules, we compared the Ends ratio profiles (Materials and Methods) obtained at different times (Figure 2F, Figures S6C and S7C). At T0, the DNA molecule had a curved region slightly upstream the TSS, in good agreement with the theoretical curvature profile predicted for the canonical pitch value 10.5 bp **(** Figure 1D). When time proceeded, the position of the curvature apex shifted towards the TSS. When comparing the curvature profiles obtained at later time T8 (Figure 2F and Figure S6C), with the theoretical profiles predicted when changing the pitch value (Figure 1D), we showed that the observed shift in the curvature apex towards the TSS could be mimicked by artificially increasing locally the pitch to values ∼11–11.5 bp. Thus, the observed dynamical evolution of the curvature properties of the DNA molecule in the TATA box and TSS regions would be the signature of some local unwinding of the DNA double helix, likely weakening the thermal requirement for strand separation (*i.e.* lowering ΔG at the TSS, see Figure 2G) and ultimately leading to base-pair opening at the required position as suggested by sharp decrease observed on the height profile in the TSS region (see Figure S7B).

### Mapping of functional and architectural proteins along the IL2RA promoter by AFM

Having examined the intrinsic structural properties of the IL2RA promoter, we next investigated the role of these properties in the formation of DNA-protein complexes.

#### TBP-TFIIB binding

Electrophoretic mobility shift assays (EMSAs) were performed on the IL2RA promoter with the presence of TBP and TFIIB GTFs (Materials and Methods and Figure S1A). When TBP and TFIIB were incubated with the IL2RA promoter, we detected a (upper) band corresponding to the TBP-TFIIB-IL2RA complex (lane 3). A similar but less intense band was also detected without TFIIB (lane 2) as the signature of the less stable TBP-DNA complex. However, this band disappeared when TBP was not added to the reaction mixture (lane 4), thereby confirming that TFIIB cannot specifically bind to DNA without the presence of TBP. We corroborated the high affinity of IL2RA promoter for GTF binding by visualizing, by AFM, the binding of TBP-TFIIB TFs on the 563 bp IL2RA fragment (Figure 3B, right panels). From the analysis of N = 50 oriented DNA molecules loaded by TBP-TFIIB GTFs, we found that these factors preferentially bind to the putative functional TATA box (Figure 3B, left panel). The fact that the TATA box region corresponds to curved DNA was previously argued as favoring the binding of these TFs as the first step of PIC formation [40]. A similar observation was obtained with the longest 1290 bp IL2RA fragment (data not shown).

#### Nucleosome assembly

We also investigated nucleosome assembly on the 898 bp IL2RA DNA fragment (Materials and Methods) with the specific goal of comparing nucleosome positioning with GTF binding in this constitutively repressed promoter region [19]. From the analysis of N = 100 oriented DNA molecules loaded by a single nucleosome (Figure 3C, right panels), we observed some lack of nucleosome positioning from −200 bp to −80 bp upstream the TSS (Figure 3C, left panel), exactly where the DNA molecule was predicted to be intrinsically supercoiled both theoretically (Figure 1) and experimentally (Figure 2). This nucleosome free region corresponds to some excluding energy barrier [19] that is coded in the DNA sequence and which further conditions the positioning of a nucleosome at (i) its left foot encompassing the PRRI region and (ii) its right foot on the TATA box and TSS (Figure 3C, left panel). Our AFM study therefore evidenced the presence of an inhibitory nucleosome on the TATA box and TSS, *i.e.* at the critical location of PIC formation, a point that was observed *in vivo* in unstimulated T-cells [41].

#### HMGA1 protein binding

The PRRII region was early on recognized [46] as a target site for HMGA1, a key player in the regulation of inducible transcription of the IL2RA gene. When further investigating the binding of HMGA1 proteins on the 898 bp IL2RA DNA fragment (Materials and Methods and Figure S1B), we observed a preferential binding in the region of intrinsically supercoiled DNA (Figure 3D, right panels) that encompasses PRRII (Figure 3D, left panel) and where nucleosomes formation was shown to be disfavored (Figure 3C) leaving DNA accessible to other protein binding factors. Note however some slight overlap with the inhibitory nucleosome positioned on the TATA box and TSS (see Discussion).

## Discussion

### What controls the TSS until transcription initiation?

The melting of DNA at the correct places is essential to recruit RNA Pol II and to initiate gene transcription. This opening is known to be directed by the helicase activity of TFIIH proteins [47] but the intrinsic structural and mechanical properties of DNA also play an important role. Thus, although a very low Gibbs free energy ΔG value in the TSS vicinity would be advantageous because less energy would be needed to open the double helix, it would increase the risk of uncontrolled melting. Actually, opening location is likely to result from multiple non-exclusive causes. In the IL2RA promoter region, the region of less thermal stability (very low ΔG) extends over the PRRII control element (Figure 2G) where the architectural HMGA1 TFs and Elf-1 are expected to bind and to regulate inducible transcription of the IL2RA gene [39]. The increased stability observed inward the TSS is indicative of the specificity of transcription initiation since more energy would be required to open DNA. Interestingly, similar ΔG profiles with the TSS located in a ramp between an upstream low ΔG region and a rather high ΔG region at the 5′-end of the gene, are shared by the ribosomal gene promoters [5] and by the yem-α TATA-less RNA Pol II promoter [48]. The DNA structure and in particular the supercoiled conformation theoretically predicted (Figure 1) and experimentally observed (Figure 2) in the PRRII region could also help preventing DNA strand separation at the TSS. It could be argued that the supercoil found on the IL2RA molecules (Figure 2, Figures S6 and S7) was not intrinsic to the DNA sequence but induced by the interaction with the substrate. Hopefully, several experimental observations allowed us to reject this objection without denying the influence of the substrate. First, formation of this supercoil was independent of the size of the IL2RA DNA fragment (Figure 2 and Figure S2F and G). Second, for all the IL2RA molecules so far tested (Figure 2, and Figures S6 and S7), the supercoil was found exactly at the same position which coincided with our theoretical modeling prediction (Figure 1). Third, time-lapse studies confirmed that the imaged DNA molecules were sufficiently free to move relative to the substrate.

In addition to these thermodynamic and structural features, we observed, consistently with a previous *in vivo* study [41], that an inhibitory nucleosome was positioned on the PRRII control region, the TATA box and the TSS (Figure 3C). This critically positioned nucleosome, in addition to preventing both TBP and TFIIB binding and in turn PIC formation on the TATA box and Elf-1 binding on PRRII, might also take part in preventing uncontrolled DNA melting by maintaining stably closed the double-helix in the vicinity of the TSS. Furthermore, we showed that another nucleosome was critically positioned on the 5′ upstream PRRI control region as an obstacle to the binding of NF-κB, SRF and Sp1 TFs.

### TSS is poised to open

**Figure 4:**
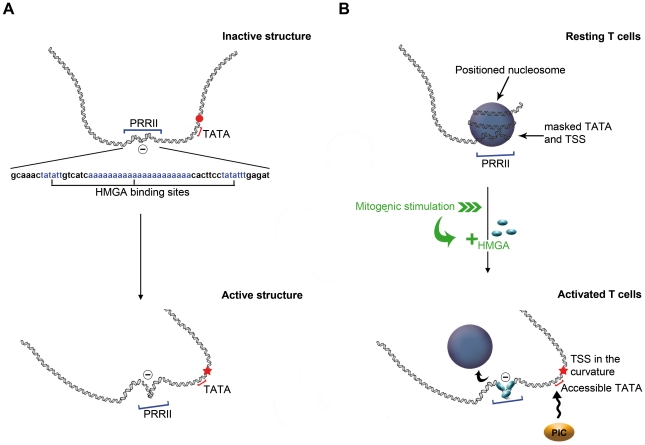
Tentative model for the activation of IL2RA gene expression. (A) Schematic representation of structural changes induced by the dynamics of a negative supercoil (circled minus) in a region containing the PRRII element (blue bracket) on naked DNA. The supercoil twisting (or DNA untwisting) allows the repositioning of the TSS from a straight portion keeping it closed (red dot), to the curvature apex of the molecule, thus favoring the melting of this region (red star). A red bracket indicates the position of the TATA box. (B) Model of the IL2RA promoter before (resting cell) and after (activated T cells) activation. The upper panel depicts the centrally positioned nucleosome masking part of PRRII (blue bracket), the TATA box and the TSS region, preventing both the PIC formation and Elf-1 binding, and keeping closed the TSS region. After activation, the binding of three HMGA molecules on PRRII induces a negative torsional stress that contributes to the remodeling and/or the ejection of the nucleosome, and allows the binding of the PIC and of Elf-1 on the TATA box (red bracket) and PRRII, respectively (lower panel). The TSS (+1, red star) is at the DNA curvature apex.

If the increase in thermal stability and the intrinsic supercoiled structure are advantageous to prevent uncontrolled DNA melting in the TSS region (Figure 2G), they should not be obstacles to the opening of the double helix at the time of transcription initiation. Several other structural characteristics of the DNA molecule are known to facilitate base-pair opening including topologically forced DNA curvature that weakens the thermal requirement for strand separation [49]. This is explained by a simultaneous lowering of the stacking energy and by the accumulation of energy within the sugar phosphate backbone which may be further released to open the double helix. In this *in vitro* study, we showed that the overtwisting of the intrinsic supercoiled structure located in the PRRII region was accompanied by the progressive positioning of the curvature apex at the TSS (Figure 2G and F and Figure S6), with the probable thermodynamic consequence of facilitating a localized melting of the double helix [49], [50]. Since this dynamical evolution was observed in the absence of initiation factors, local torsional stress likely induced by the extensive interaction with the charged mica surface, was apparently sufficient to unwind DNA. A sequence-dependent preference to the opening of the double helix at or near the TSS has been reported in an experimental study of different types of promoters [51]. The present AFM study suggests that the intrinsic structure of the IL2RA promoter is a key actor in the activation process of the IL2RA gene (Figure 4A). *In vivo* probably under the action of architectural TFs, conformational changes induced by DNA unwinding are likely to direct the pre-melting reaction at the prescribed TSS locus. In addition, the modifications detected in DNA flexibility and curvature properties in the TATA box vicinity are likely to facilitate the binding of TBP and TFIIB GTFs at this particular region [40] (Figure 3B) thereby promoting PIC formation.

### PRRII control region: an adequate substrate for HMGA1 proteins

The functional role of PRRII in the regulation of the IL2RA gene expression was first evidenced by a mutation within this region inhibiting its transcription [46]. Binding of HMGA1 (Figure 3D) on an intrinsic supercoiled structure was revealed both by AFM experiment (Figure 2) and theoretical modeling (Figure 1). Actually the PRRII sequence is characterized by an anomalous high A+T content (AT-tracts) and the intrinsic supercoil possibly results from the regular spacing of the AT-tracts with a periodicity slightly out of phase with the mean helical pitch. PRRII sequence contains three AT-tracts, separated by 6 and 7 bp, the longest one being 19 bp long, corresponding to three potential binding sites with different affinities for HMGA1. HMGA1 contains AT-hook domains allowing its binding to the minor groove of AT-rich sequences; high affinity actually requires multiple properly phased AT-tracts [52] with spacing <10 bp. Interestingly, the AT-tracts in PRRII are separated by less that 10 bp which suggests that the observed negative supercoiled DNA structure is the source of specificity for HMGA1 positioning and high affinity binding. Importantly AFM further revealed that the PRRII region is drastically depleted in histone octamer binding [19] (Figure 3C). Because of their relative rigidity, multiple poly(dA:dT) stretches are known to be poorly incorporated into nucleosomes *in vivo* [4], [8], [16]. The presence of A-tracts in the PRRII region impairs the structural distortions required for nucleosome formation, leaving some room for HMGA1 binding.

### A scenario for transcription activation

Indeed, HMGA1 binding is likely to be actively involved in the remodeling of the inhibitory nucleosome in response to T-cells activation [53]. Besides HMGA1 binding, additional processes, including histone modifications, must contribute to remodel the repressive nucleosome. Our results suggest that this remodeling might have some dynamical and structural origin. The observed dynamical evolution of the intrinsic left-handed supercoiled structure in the IL2RA promoter region could act as a lever arm helping nucleosome remodeling and/or ejection. This attractive picture is consistent with the experimental observation that when binding to a closed circular DNA at a high protein/DNA ratio, HMGA1 induces a negative torsional stress which, by accumulation, results in a negative supercoil [53]. In our AFM study, as a substitute to HMGA1 action, the electrostatic interaction of free DNA with a positively charged mica surface could induce some dynamical overtwisting of the supercoil that ultimately might destabilize the nucleosome positioned on the TATA box and TSS. We propose the following scenario for IL2RA transcription activation. In a physiological context, resting T-cells contain low but detectable amounts of HMGA1, insufficient to induce nucleosome ejection. Following activation, the increase in concentration of this protein and its binding on its three specific sites (AT-tracts) could act as a switch by giving the torsional stress necessary to promote remodeling of the inhibitory nucleosome, and in turn Elf-1 fixation. In addition, the propagation of this torsional stress (DNA unwinding), accompanied by a shift of the DNA curvature apex towards the TSS, would also facilitate DNA melting and the recruitment of the RNA Pol II at the TATA box.

The importance of supercoiled structures in the regulation of transcription process has been the subject of increasing interest. For example, in prokaryotes, the ilvP_G_ gene of *E. coli* requires an intrinsically supercoiled DNA template located at the promoter to allow the binding at position −92 bp of the integration host factor (IHF). This peculiar structure was shown to mediate the translocation of superhelical energy, to facilitate stabilization in the −10 bp promoter region, and to activate transcription by increasing the rate of open complex formation [54]. These mechanisms are likely to be important for the regulation of transcription in eukaryotes as well. In *S.cerevisiae* CUP1 gene, negatively supercoiled DNA templates apparently allow RNA Pol II entry even in the absence of the TBP [55]. Supercoiled DNA templates were further shown to be required for enhancer-mediated *in vitro* regulation of the chicken β^A^-globin and mouse T-cell receptor genes [56]. Negative DNA superhelicity is also involved in the basal level of transcription of the c-myc gene [57]. Very much like the PRRII control region of the IL2RA gene promoter, all these regions with peculiar supercoiled DNA properties appear as ideal target for DNA-binding proteins.

In conclusion, by combining DNA modeling and AFM imaging in liquid, we investigated the structure and dynamics of the IL2RA gene promoter and suggested the following model for the activation of gene expression (Figure 4B): a) HMGA1 multiple binding to specific sites in the PRRII region, b) remodeling of the inhibitory nucleosome positioned on the TATA box and TSS via torsional stress propagation, c) timely DNA melting at a specific region via local DNA curvature enhancing and d) recruitment and binding of the PIC. Further studies might be required to determine whether this sequence-specific structural and dynamical activation scenario is peculiar to the human IL2RA gene promoter or can be extended to other inducible RNA Pol II-dependent promoters.

## Supporting Information

Figure S1 **Analysis of the complexes of transcription factors and DNA by electrophoretic mobility shift assay.** (A) EMSA performed with (from left to right): 563 bp IL2RA naked DNA (lane 1), 563 bp IL2RA DNA and TBP (lane 2), 563 bp IL2RA DNA, TBP and TFIIB (lane 3), 563 bp IL2RA DNA and TFIIB (lane 4) and DNA ladder 100 bp (lane 5). Bands in the gel correspond only to naked DNA and DNA-protein complexes except for the DNA ladder (lane 5). (B) EMSA performed with the 898 bp IL2RA DNA fragment and HMGA for increasing [HMGA1]/[DNA] ratio. The molar ratios of HMGA to DNA were increased from 0 to 3 (0, 1, 2, 3) for lanes 1 to 4.
(TIF)

Figure S2 **AFM imaging of the IL2RA gene promoter region.** (A) Large scale (1×1 µm) AFM imaging in liquid of 1290 bp fragments in aqueous buffer. AFM imaging in liquid of 1290 bp fragments (B, C), 898 bp fragments (D, E) and 563 bp fragments (F, G).
(TIF)

Figure S3 **AFM image treatment and molecule orientation.** (A-A’’’) Molecule path skeletonization from an AFM image of the 1290 bp human IL2RA fragment. (B-B’’’) Illustration of the methodology used to orient the IL2RA fragment (19): the pattern of height variation along the axis of the fragment (red) is compared to the calculated pitch variation (black) obtained when using the Bolshoy *et al*. [44] DNA coding table. The reproducibility in AFM topography measurement and its correlation with sequence-dependent pitch modeling allowed us to orient a significant proportion of the IL2RA molecules imaged by AFM. Indeed there is a significant height difference between the 5′ end (low) and 3′ end (high), that turn out to be very useful to orient the underlying DNA sequence. The efficiency of this method was confirmed *a posteriori* by the very accurate positioning of the TBP-TFIIB GTFs at sites corresponding to the TATA sequence (Figure 3B) as expected for a first step of PIC formation.
(TIF)

Figure S4 **AFM imaging of GTF, nucleosome and HMGA1 positioning on the IL2RA gene promoter.** (A) AFM imaging of TBP-TFIIB positioning along the 563 bp IL2RA fragment (left panels) and topological profile along the molecule path skeleton (right panels). (B) AFM imaging of mononucleosome positioning along the 898 bp IL2RA fragment (left panels) and topological profile along the molecule path skeleton (right panels) (see Ref. [19]). (C) AFM imaging of HMGA1 positioning along the 898 bp IL2RA fragment (left panels) and topological profile along the molecule path skeleton (right panels).
(TIF)

Figure S5 **Prediction of the DNA structure of a control DNA fragment.** (A) AFM images of representative 976 bp pBR control fragments (Materials and Methods) in aqueous buffer. (B) Theoretical analysis of the pBR sequence. The diagrammatic (upper plane) and spectral (lower graph) analyses do not show the presence of supercoiled DNA, as previously revealed for the IL2RA sequence (Figure 1). Window size is 150 bp, step 1 bp, analyzed phasing pitch varies from 5 to 15 bp with step 1 bp. (C) pBR molecule path skeletonization. (D) Height measurement along the red path in (C). (E) Standard deviation of height measurements from 97 pBR molecules (black); for comparison is shown the height standard deviation from 100 IL2RA molecules (blue).
(TIF)

Figure S6 **Time-lapse AFM imaging of the IL2RA gene promoter region.** (A) AFM images of a representative 1290 bp IL2RA fragment in aqueous buffer. (B) Time lapse AFM images of this fragment in the enlarged white box in (A) at 0, 4 min, 6 min and 8 min after beginning the observation. The position of the TSS is indicated by a red star. At 4 min, a negative supercoil was observed. In the t = 0 image, the white arrow indicates the position where the supercoiled structure will form. (C) Corresponding curvature profiles, expressed as Ends ratios, along the fragment (window 150 bp, step 1 bp). The position of the TSS is indicated by a vertical blue line.
(TIF)

Figure S7 **Time-lapse AFM imaging of the IL2RA gene promoter region.** (A) AFM images of a representative 1290 bp IL2RA fragment in aqueous buffer.(B) Height variation along the DNA molecule path skeleton. (C) Time lapse AFM images of this fragment in the enlarged white box in (A) at 0, 4 min, 6 min and 8 min after beginning the observation. The position of TSS is indicated by a red star. For this molecule the supercoil is already present at t = 0. (D) Corresponding curvature profiles, expressed as Ends ratio, along the fragment (window 150 bp, step 1 bp). The position of the TSS is indicated by a vertical blue line.
(TIF)

Figure S8 **AFM imaging of DNA loop.** (A) Enlarged AFM image of the loop formed on the 1290 bp IL2RA fragment (Figure 2). (B) Height variation along the four colored lines in (A); the reference red line is drawn to allow relative positioning of the four topographic curves. (C) Schematic drawing of the corresponding loop. (D) Enlarged AFM image of a loop formed on the 900 bp IL2RA fragment. (E) Height variation along the four colored lines in panel (D). (F) Schematic drawing of the corresponding loop.
(TIF)

Figure S9 **AFM image of a DNA loop.** (A) Size distribution of DNA loops visualized on 21 images of the 1290 bp and 900 bp IL2RA fragments. (B) AFM images showing a DNA loop on the 1290 bp fragment. (C) AFM images showing a DNA loop on the 898 bp fragment.
(TIF)

Table S1 List of primers used during the PCR reaction for the amplification of the 1290 bp, 898 bp and 563 bp IL2RA DNA fragments.
(DOC)

Video S1 Consecutive AFM images of the IL2RA 1290 bp (Figure 2) during continuous scanning in the aqueous buffer at the frequency of 1 frame every 37 seconds.
(MOV)

Video S2 Same as Video S1 for the 1290 bp IL2RA (Figure S6).
(MOV)

Video S3 Same as Video S1 for the 1290 bp IL2RA (Figure S7).
(MOV)
