# Supplementary figures and images for: Mechanics of the IL2RA Gene Activation Revealed by Modeling and Atomic Force Microscopy
Source: PLoS One. 2011 Apr 13;6(4):e18811. doi: 10.1371/journal.pone.0018811 (PMC3076448; doi:10.1371/journal.pone.0018811)

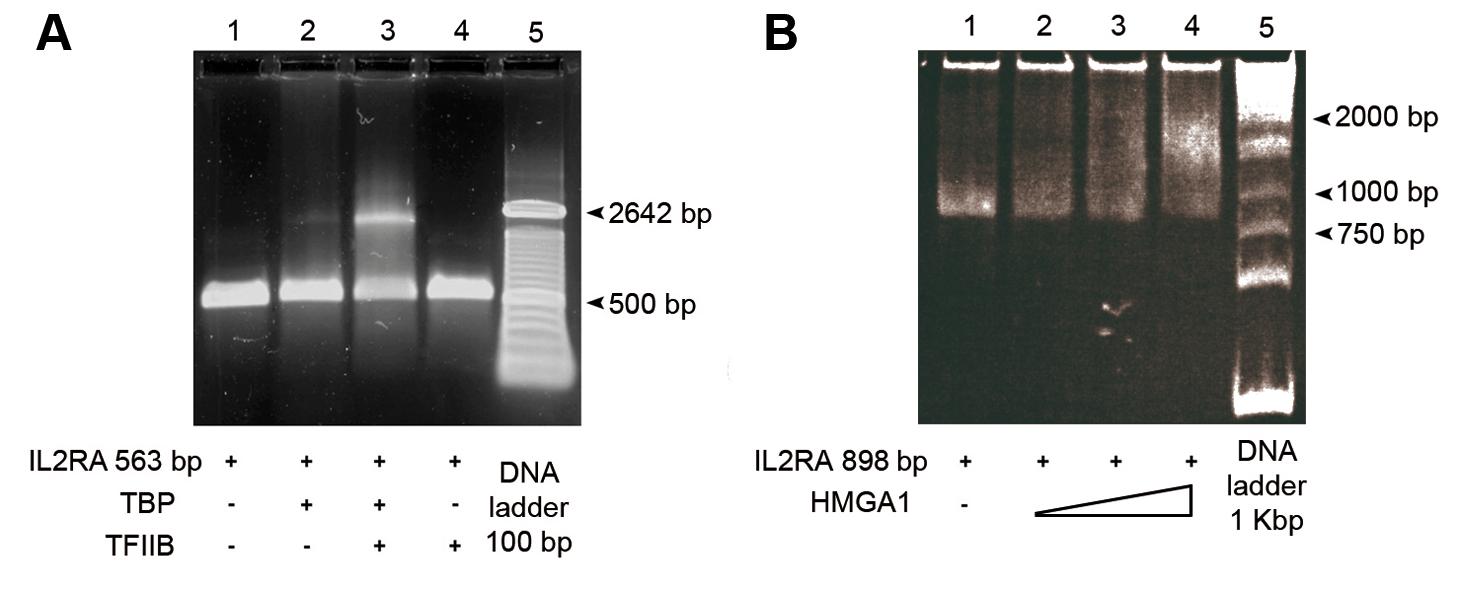

Supplement: Figure S1 — Analysis of the complexes of transcription factors and DNA by electrophoretic mobility shift assay. (A) EMSA performed with (from left to right): 563 bp IL2RA naked DNA (lane 1), 563 bp IL2RA DNA and TBP (lane 2), 563 bp IL2RA DNA, TBP and TFIIB (lane 3), 563 bp IL2RA DNA and TFIIB (lane 4) and DNA ladder 100 bp (lane 5). Bands in the gel correspond only to naked DNA and DNA-protein complexes except for the DNA ladder (lane 5). (B) EMSA performed with the 898 bp IL2RA DNA fragment and HMGA for increasing [HMGA1]/[DNA] ratio. The molar ratios of HMGA to DNA were increased from 0 to 3 (0, 1, 2, 3) for lanes 1 to 4. (TIF) [file pone.0018811.s001.tif]

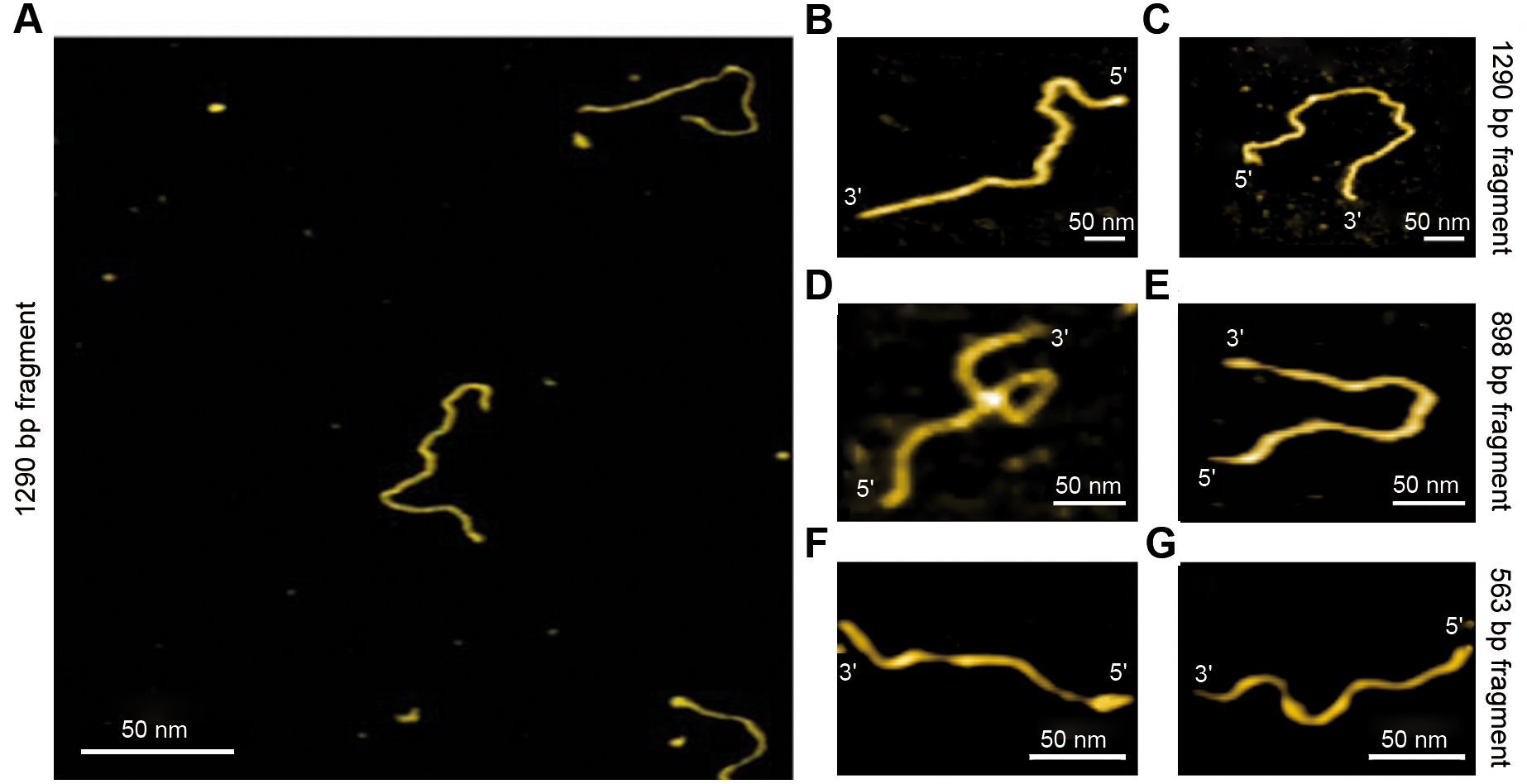

Supplement: Figure S2 — AFM imaging of the IL2RA gene promoter region. (A) Large scale (1×1 µm) AFM imaging in liquid of 1290 bp fragments in aqueous buffer. AFM imaging in liquid of 1290 bp fragments (B, C), 898 bp fragments (D, E) and 563 bp fragments (F, G). (TIF) [file pone.0018811.s002.tif]

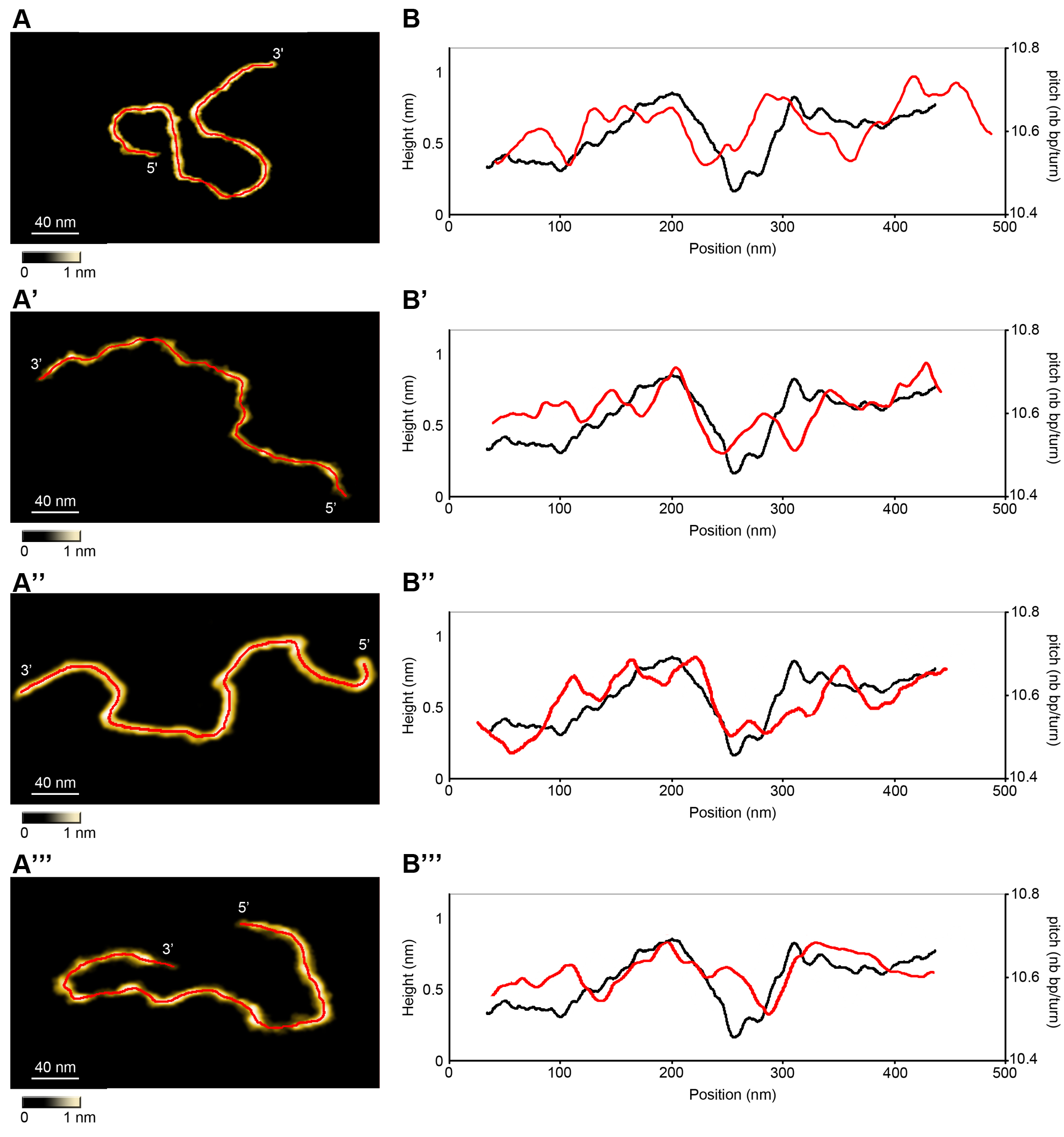

Supplement: Figure S3 — AFM image treatment and molecule orientation. (A-A’’’) Molecule path skeletonization from an AFM image of the 1290 bp human IL2RA fragment. (B-B’’’) Illustration of the methodology used to orient the IL2RA fragment (19): the pattern of height variation along the axis of the fragment (red) is compared to the calculated pitch variation (black) obtained when using the Bolshoy et al. [44] DNA coding table. The reproducibility in AFM topography measurement and its correlation with sequence-dependent pitch modeling allowed us to orient a significant proportion of the IL2RA molecules imaged by AFM. Indeed there is a significant height difference between the 5′ end (low) and 3′ end (high), that turn out to be very useful to orient the underlying DNA sequence. The efficiency of this method was confirmed a posteriori by the very accurate positioning of the TBP-TFIIB GTFs at sites corresponding to the TATA sequence (Figure 3B) as expected for a first step of PIC formation. (TIF) [file pone.0018811.s003.tif]

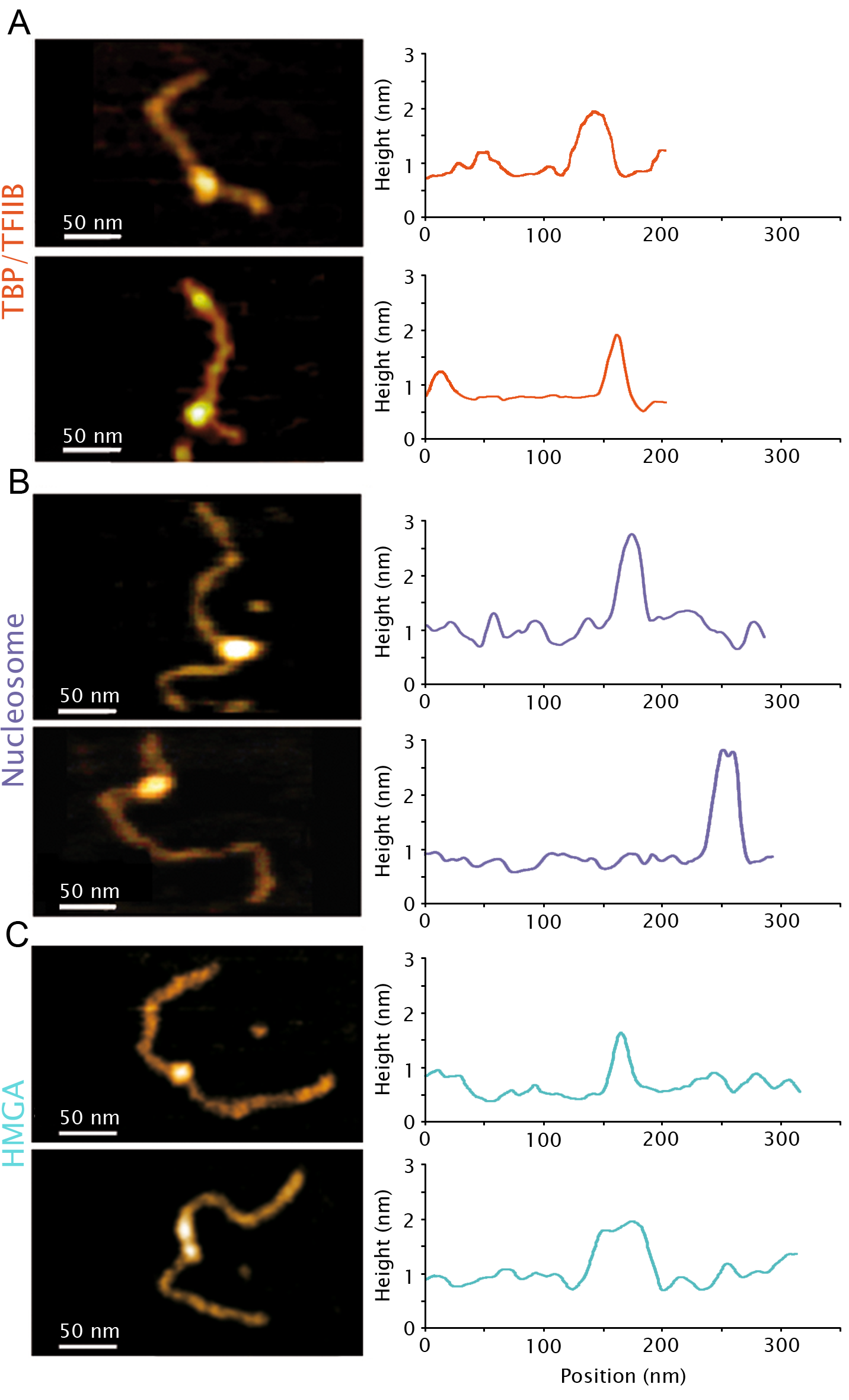

Supplement: Figure S4 — AFM imaging of GTF, nucleosome and HMGA1 positioning on the IL2RA gene promoter. (A) AFM imaging of TBP-TFIIB positioning along the 563 bp IL2RA fragment (left panels) and topological profile along the molecule path skeleton (right panels). (B) AFM imaging of mononucleosome positioning along the 898 bp IL2RA fragment (left panels) and topological profile along the molecule path skeleton (right panels) (see Ref. [19]). (C) AFM imaging of HMGA1 positioning along the 898 bp IL2RA fragment (left panels) and topological profile along the molecule path skeleton (right panels). (TIF) [file pone.0018811.s004.tif]

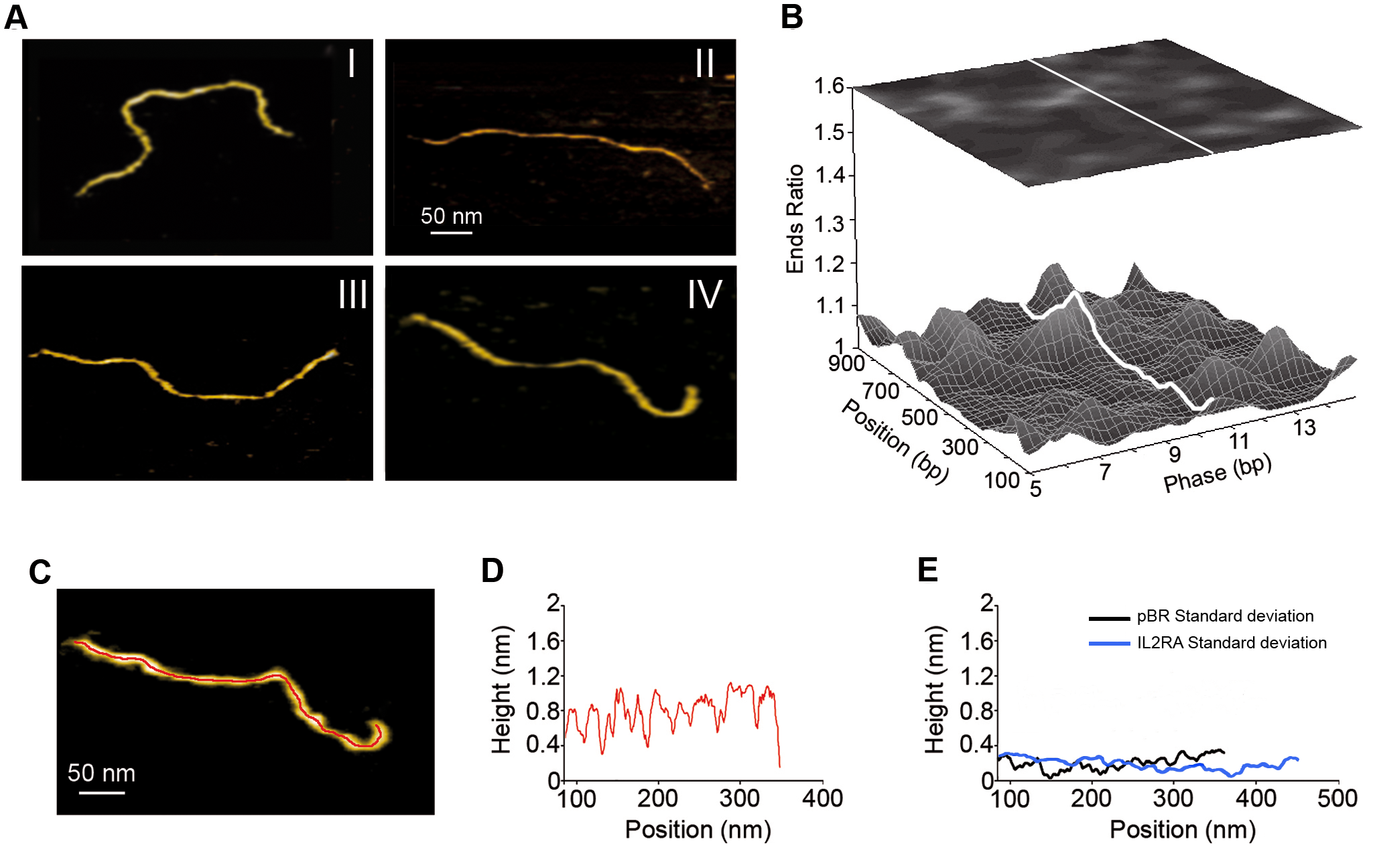

Supplement: Figure S5 — Prediction of the DNA structure of a control DNA fragment. (A) AFM images of representative 976 bp pBR control fragments (Materials and Methods) in aqueous buffer. (B) Theoretical analysis of the pBR sequence. The diagrammatic (upper plane) and spectral (lower graph) analyses do not show the presence of supercoiled DNA, as previously revealed for the IL2RA sequence (Figure 1). Window size is 150 bp, step 1 bp, analyzed phasing pitch varies from 5 to 15 bp with step 1 bp. (C) pBR molecule path skeletonization. (D) Height measurement along the red path in (C). (E) Standard deviation of height measurements from 97 pBR molecules (black); for comparison is shown the height standard deviation from 100 IL2RA molecules (blue). (TIF) [file pone.0018811.s005.tif]

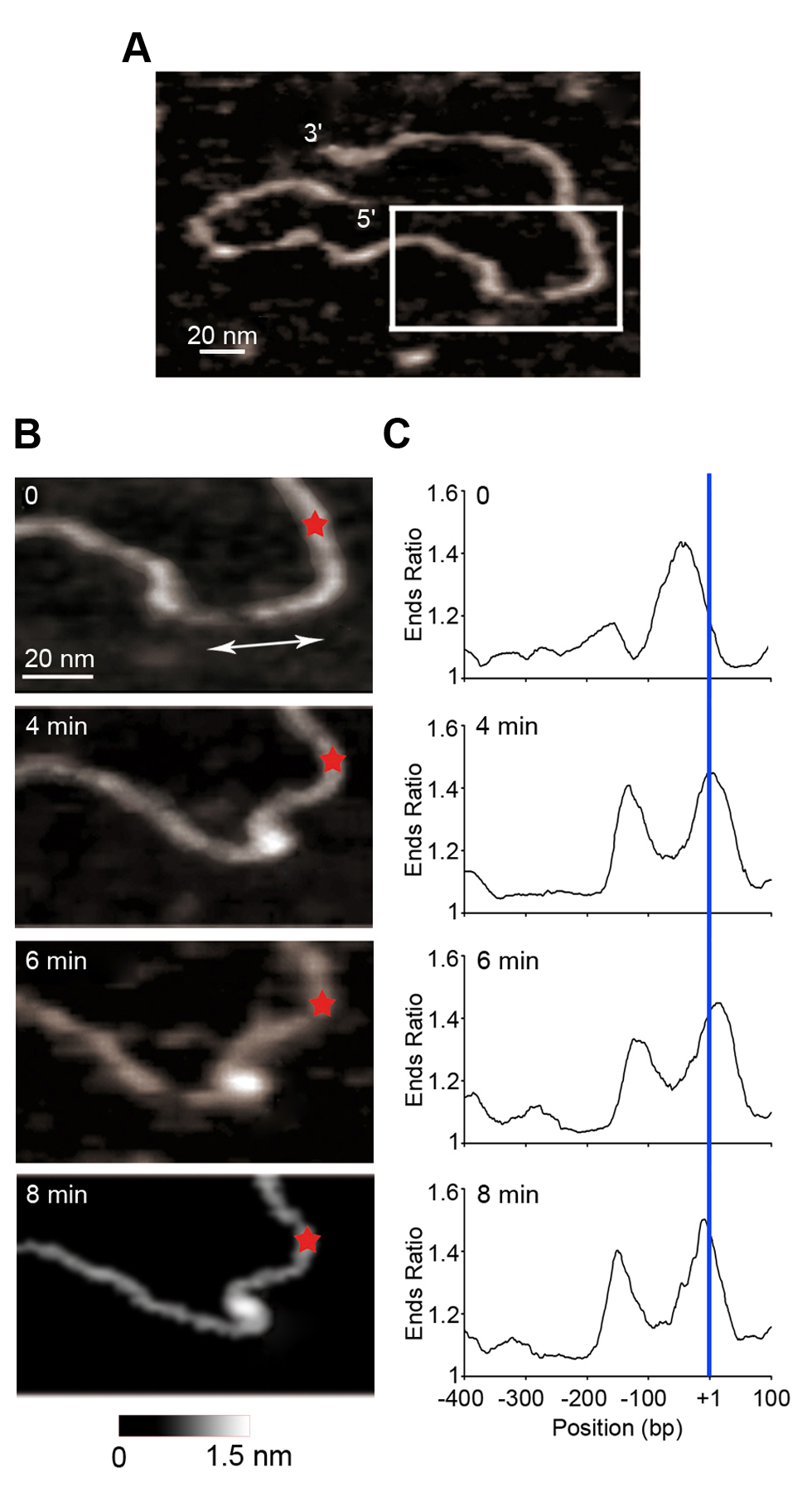

Supplement: Figure S6 — Time-lapse AFM imaging of the IL2RA gene promoter region. (A) AFM images of a representative 1290 bp IL2RA fragment in aqueous buffer. (B) Time lapse AFM images of this fragment in the enlarged white box in (A) at 0, 4 min, 6 min and 8 min after beginning the observation. The position of the TSS is indicated by a red star. At 4 min, a negative supercoil was observed. In the t = 0 image, the white arrow indicates the position where the supercoiled structure will form. (C) Corresponding curvature profiles, expressed as Ends ratios, along the fragment (window 150 bp, step 1 bp). The position of the TSS is indicated by a vertical blue line. (TIF) [file pone.0018811.s006.tif]

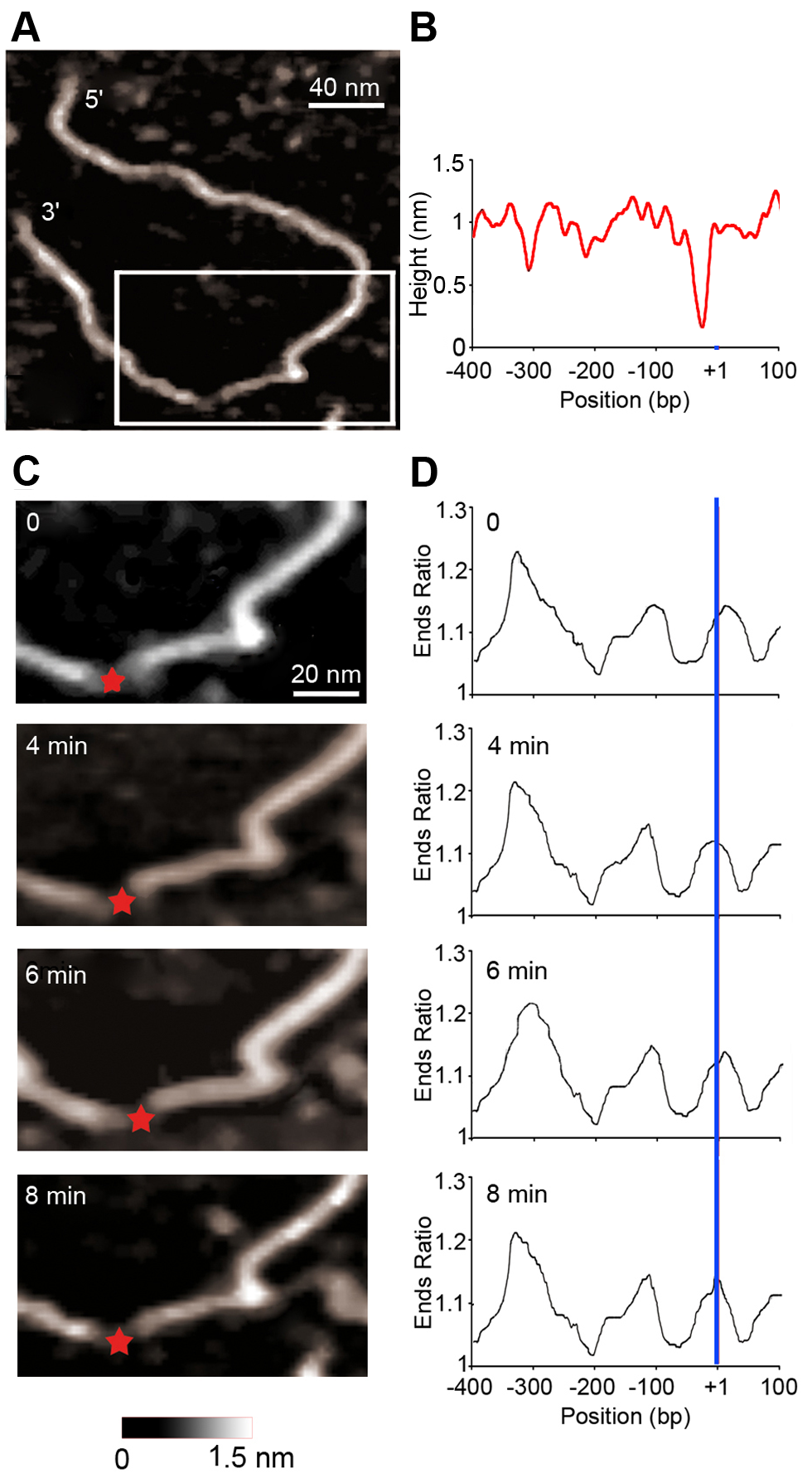

Supplement: Figure S7 — Time-lapse AFM imaging of the IL2RA gene promoter region. (A) AFM images of a representative 1290 bp IL2RA fragment in aqueous buffer.(B) Height variation along the DNA molecule path skeleton. (C) Time lapse AFM images of this fragment in the enlarged white box in (A) at 0, 4 min, 6 min and 8 min after beginning the observation. The position of TSS is indicated by a red star. For this molecule the supercoil is already present at t = 0. (D) Corresponding curvature profiles, expressed as Ends ratio, along the fragment (window 150 bp, step 1 bp). The position of the TSS is indicated by a vertical blue line. (TIF) [file pone.0018811.s007.tif]

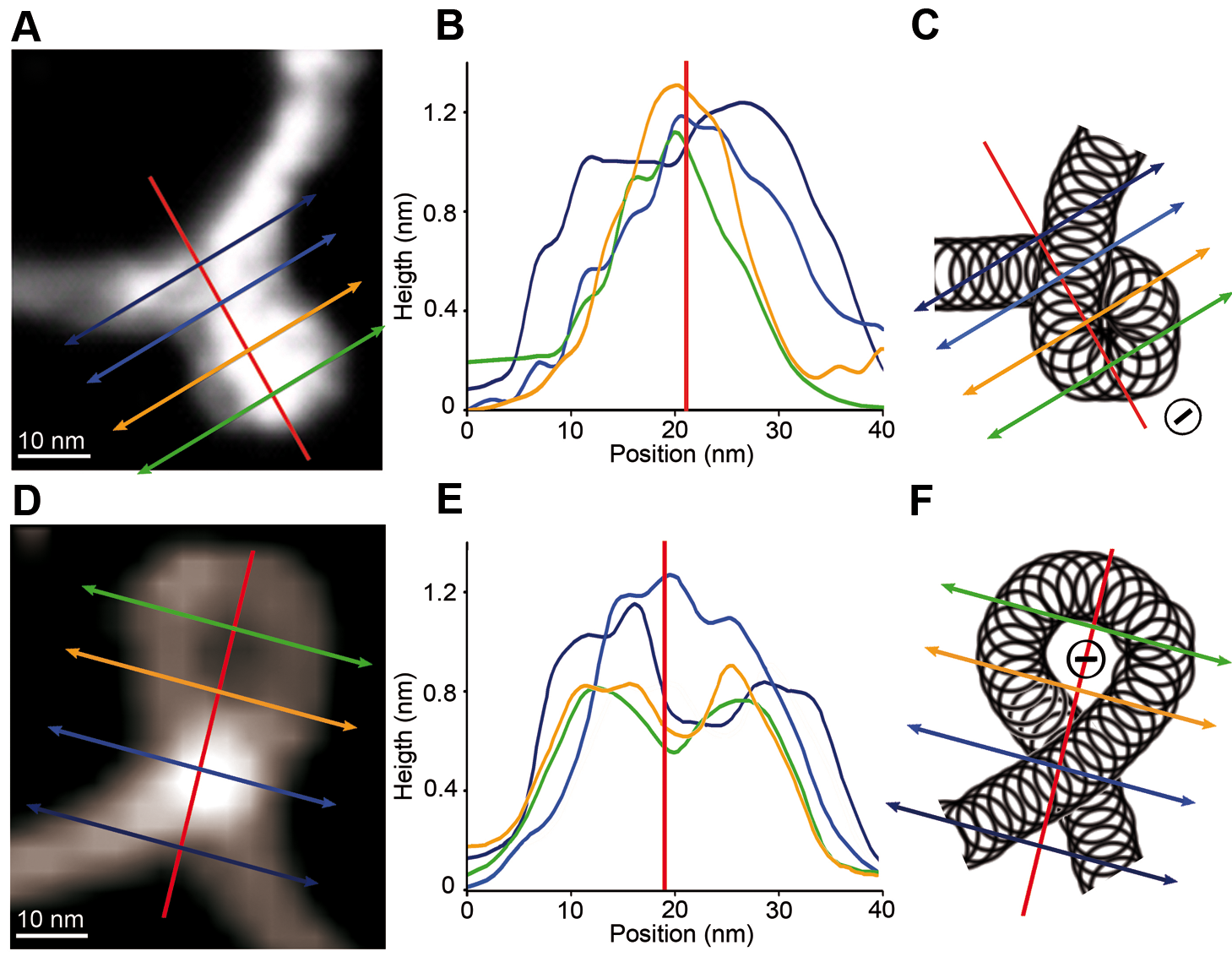

Supplement: Figure S8 — AFM imaging of DNA loop. (A) Enlarged AFM image of the loop formed on the 1290 bp IL2RA fragment (Figure 2). (B) Height variation along the four colored lines in (A); the reference red line is drawn to allow relative positioning of the four topographic curves. (C) Schematic drawing of the corresponding loop. (D) Enlarged AFM image of a loop formed on the 900 bp IL2RA fragment. (E) Height variation along the four colored lines in panel (D). (F) Schematic drawing of the corresponding loop. (TIF) [file pone.0018811.s008.tif]

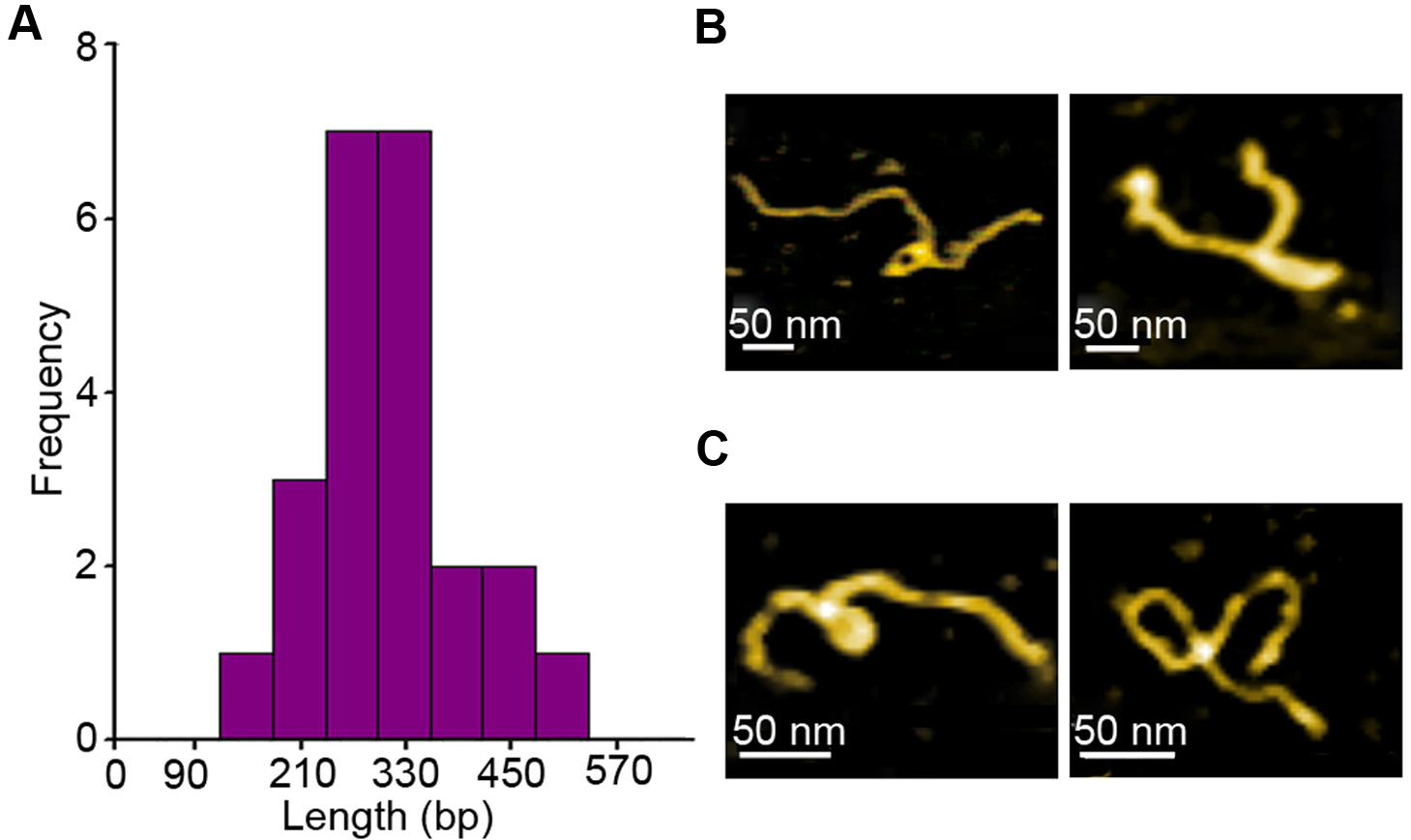

Supplement: Figure S9 — AFM image of a DNA loop. (A) Size distribution of DNA loops visualized on 21 images of the 1290 bp and 900 bp IL2RA fragments. (B) AFM images showing a DNA loop on the 1290 bp fragment. (C) AFM images showing a DNA loop on the 898 bp fragment. (TIF) [file pone.0018811.s009.tif]
