# Supplementary material for: Mechanics of the IL2RA Gene Activation Revealed by Modeling and Atomic Force Microscopy
Source: PLoS One. 2011 Apr 13;6(4):e18811. doi: 10.1371/journal.pone.0018811 (PMC3076448; doi:10.1371/journal.pone.0018811)
Supplement: Table S1 — List of primers used during the PCR reaction for the amplification of the 1290 bp, 898 bp and 563 bp IL2RA DNA fragments. (DOC) [file pone.0018811.s010.doc]

| **IL2RA DNA fragment** | **Forward primer** | **Reverse primer** |
| --- | --- | --- |
| 1290 bp | 5’-GTGTTTTGGAACCCTGAATTCC-3’ | 5'-GCTATTTCAGGCTCTCTTGACA-3' |
| 898 bp | 5’-TGACAATGCACTTTCAGGAGC-3’ | 5'-CAACTCCCTTCTTGGAACCATC-3' |
| 563 bp | 5’-GGATCCTTCAGTTCGCCGCAT-3’ | 5’-GTCAGCCTCTTTTTGGCATCG-3’ |
